# Report of a Case of Typhoid Fever with Unusual Features

**Published:** 1905-10

**Authors:** G. Pope Huguley

**Affiliations:** Atlanta, Ga.


### REPORT OF A CASE OF TYPHOID FEVER WITH UNUSUAL FEATURES.

By G. POPE HUGULEY, M. D., Atlanta, Ga.

Miss. L. B. L. aet. 23, resident of Atlanta. Patient first seen at 10 o'clock, evening of August 26th, 1905. History of two previous attacks of typhoid, the last one two years ago. Had been in Birmingham, Alabama, during the three weeks preceding. Felt unwell and not able to work. Persistent and violent headache. Returned to Atlanta on Tuesday, August 22d—afternoon of that day chill followed by rise in temperature. Continued at work, however, until evening of August 26th. Condition at time first seen, temperature 103, pulse 112. Nausea, vomiting frequently, large quantities of yellow fluid mixed with mucus.

Patient removed to a private infirmary on the 28th. Temperature on admission (10:20 a. m.) 101 4-5, pulse 96. At 2:30 and again at 5 p. m., temperature 101 1-5, pulse 100, respiration 30, slight abdominal distention. Vomiting continued. Delirious at times.

Capsule containing mercury, guiacol-carb. and sodium bicarbonate followed with saline ordered. After free purging, vomiting practically ceased.

Thereafter her daily temperature ranged from 103 3-5 in the morning to 104 3-5 in the afternoon, showing very slight variations, remaining almost steadily at 103 3-5. Cold sponging with cold pack was ordered when temperature was above 102. Acetozone internally produced vomiting almost invariably, this, after thorough trial, was discontinued. Gave capsule containing beta-naphthol, guiacol-carb. and quinine-bisulph. and solution of nitro-hydrochloric acid, dilute with essence of pepsin. Diet of egg albumen, soups, broths, beef tea, panopepton gelatin. Milk with lime water discontinued after one day's use. Curds appeared in the stool and flatulence increased. High enemata of normal saline solution, one pint twice daily. It was noticed that the cold sponging and pack not only failed to reduce the temperature, but the after bath temperature, taken one hour later, uniformly showed an increase from one-half to one degree. Hot sponging with hot pack was then ordered with the same result. The patient reacted better to the cold, so cold was again ordered, in addition, phenacetine grs. three, whiskey oz. one-half was ordered given when the after bath temperature registered 103 or more. This, having no effect, was discontinued. The pulse was at all times good, volume full and regular, only once showing slight dicrotism, that lasting for a short time only. 118 was the highest recorded. The respiration was regular, ranging from 22 to 30. There was no diarrhea. The condition of the pulse and respiration did not necessitate the use of stimulants at any time.

On September 2d Dr. W. C. Jarnagin saw the case with me, concurring in the diagnosis and treatment. The temperature on the evening of the seventh day in the infirmary was 103 2-5, at twelve midnight registered 102 1-10. This was the first decline since being admitted, and the only time under 103 since admission.

The morning of the eighth day in the infirmary at 6 o'clock a. m., the temperature was 100 3-5, pulse 92, respiration 25. At 6 o'clock p. m. temperature 101 3-5, pulse 82, respiration 22. Delirium disappearing. On the ninth day, 6 o'clock a. m., temperature

100 1-5, pulse 80, respiration 30. Patient had comfortable night, quiet, but slept little. Six o'clock p. m., of that day her temperature registered 101, being the maximum temperature for that day. I saw the patient between six-thirty and seven p. m., she was conscious and cheerful, expressed herself as feeling much better and stronger, and expressed a de-ire to be moved to her home as soon as expedient. At two-five in the morning I was called to the phone and informed that the patient appeared to be dying. Before I could complete dressing, a second message informed me that the patient had expired. The chart shows at 9 p. m. temperature 101 2-5, pulse 90, respiration 30. At twelve, midnight, temperature 101 3-5, pulse 84, respiration 30, being given panopepton oz. one, she immediately vomited, as she had done several times previously. The nurse on duty reports that at 2 o'clock a. m. she went in to see the patient, her condition at that time good, temperature as reported to me verbally upon my arrival at the infirmary short time afterward, 101, pulse about 80. Unfortunately, this was not charted. She left the room to prepare some nourishment, on her return in less than five minutes, found patient dying. She immediately summoned the superintendent of nurses and the house physician. They reported to me that the patient gasped once or twice after they saw her, no pulsation of the heart being appreciable.

The steady, persistent, high temperature showing unusual involvement of the heat center; the absolute failure of the above described measures to reduce the temperature, on the contrary seemingly causing it to mount higher, the uniformly good character of the pulse and respiration throughout the entire illness, comparatively slight abdominal symptoms, and the sudden and wholly unexpected death, combine to make the case unusual as to these features. Post mortem could not be obtained.

---

The Newton County Medical Society organized July 25, 1905, and elected the following officers:

Dr. E. W. Ragsdale, President; Dr. P. Wilson, Vice-President Dr. W. D. Travis, Secretary and Treasurer.

Meetings will be held the first Tuesday in each month.

